# Time required for gas exchange equilibration after a change of positive end-expiratory pressure in acute respiratory distress syndrome

**DOI:** 10.1186/cc12051

**Published:** 2013-03-19

**Authors:** S Coppola, D Chiumello, F Menga, M Brioni, I Cigada, S Froio

**Affiliations:** 1 Fondazione IRCCS Ca' Granda - Ospedale Maggiore Policlinico, Milan, Italy; 2 Università degli Studi di Milano, Milan, Italy

## Introduction

Positive end-expiratory pressure (PEEP) is fundamental to prevent lung collapse in ARDS patients. A common method to titrate PEEP is to perform a PEEP test, recording the variation of cardiorespiratory parameters after a PEEP change [1]. The aim of this study was to evaluate the time-course changes of gas exchanges following a PEEP test.

## Methods

Mechanically ventilated patients (PEEP 10 cmH_2_O and TV 7 ml/kg - Baseline) were randomized to two groups: in the PEEP 15 group, PEEP was increased from 10 to 15 cmH_2_O; while in the PEEP 5 group, PEEP was decreased from 10 to 5 cmH_2_O. Arterial gas analyses were performed in both groups after 5, 15, 30 and 60 minutes from the change of PEEP.

## Results

**Figure 1:**
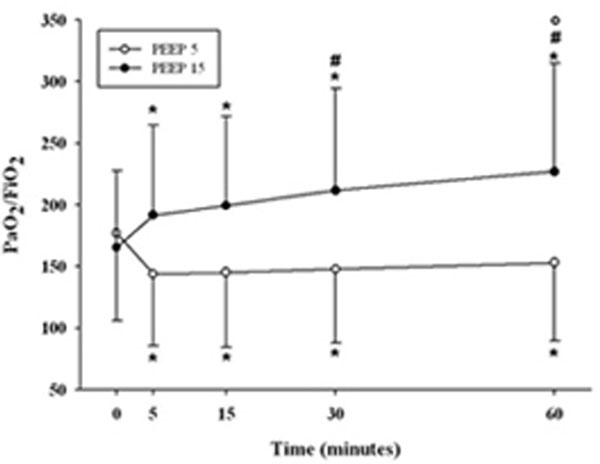
**Two-way ANOVA RM**. *P <*0.05, *versus Time 0, #versus 5 minutes, °versus 15 minutes.

**Figure 2:**
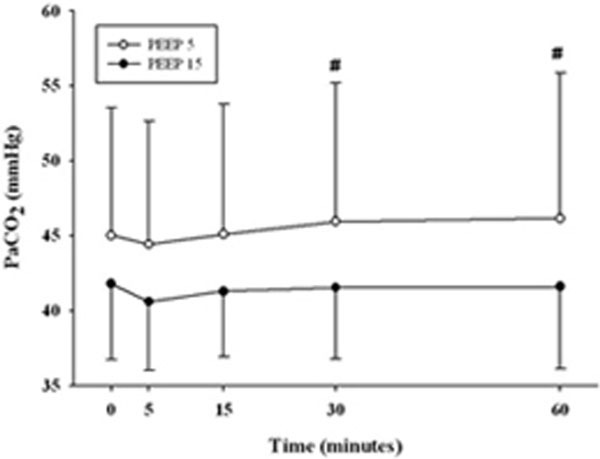
**Two-way ANOVA RM**. *P <*0.05, #versus 5 minutes.

We enrolled 44 ARDS patients: 23 in the PEEP 15 group and 21 in the PEEP 5 group. At Baseline, PaO_2_/FiO_2 _(P/F) and PaCO_2 _were similar in both groups (P/F 169.5 ± 78.8 vs. 165.4 ± 80.6; PaCO_2 _45.6 ± 8.5 vs. 41.7 ± 5.0 mmHg, PEEP 15 vs. PEEP 5, respectively). In the PEEP 15 group, P/F significantly continuously increased over time. In PEEP 5, P/F significantly decreased after 5 minutes and remained stable over time. In the PEEP 15 group, PaCO_2 _did not change within 60 minutes after PEEP increase. When PEEP was reduced (PEEP 5) PaCO_2 _remained stable for the first two steps, while at 30 and 60 minutes PaCO_2 _was significantly higher than at Baseline (Figures 1 and 2).

## Conclusion

Our data indicate that it is important in critically ill patients to allow sufficient time for the full effect of PEEP increase on oxygenation and to prevent excessive delay when P/F decrease occurs following the application of a lower level of PEEP.
